# Complexation of Anthocyanin-Bound Blackcurrant Pectin and Whey Protein: Effect of pH and Heat Treatment

**DOI:** 10.3390/molecules27134202

**Published:** 2022-06-29

**Authors:** Nurhazwani Salleh, Kelvin K. T. Goh, Mark R. Waterland, Lee M. Huffman, Mike Weeks, Lara Matia-Merino

**Affiliations:** 1 School of Food and Advanced Technology, Massey University, Palmerston North 4442, New Zealand; n.salleh@massey.ac.nz (N.S.); k.t.goh@massey.ac.nz (K.K.T.G.); 2 School of Natural Sciences, Massey University, Palmerston North 4442, New Zealand; m.waterland@massey.ac.nz; 3 The New Zealand Institute for Plant and Food Research Ltd., Palmerston North 4472, New Zealand; lee.huffman@plantandfood.co.nz; 4 AgResearch Ltd., Te Ohu Rangahau Kai, Massey University, Palmerston North 4442, New Zealand; mike.weeks@agresearch.co.nz

**Keywords:** blackcurrant pectin extract, blackcurrant polyphenols, whey protein, anthocyanin-protein interactions, pectin-protein interactions, light scattering, FTIR

## Abstract

A complexation study between blackcurrant pectin (BCP) and whey protein (WP) was carried out to investigate the impact of bound anthocyanins on pectin–protein interactions. The effects of pH (3.5 and 4.5), heating (85 °C, 15 min), and heating sequence (mixed-heated or heated-mixed) were studied. The pH influenced the color, turbidity, particle size, and zeta-potential of the mixtures, but its impact was mainly significant when heating was introduced. Heating increased the amount of BCP in the complexes—especially at pH 3.5, where 88% *w*/*w* of the initial pectin was found in the sedimented (insoluble) fraction. Based on phase-separation measurements, the mixed-heated system at pH 4.5 displayed greater stability than at pH 3.5. Heating sequence was essential in preventing destabilization of the systems; mixing of components before heating produced a more stable system with small complexes (<300 nm) and relatively low polydispersity. However, heating WP before mixing with BCP prompted protein aggregation—producing large complexes (>400 nm) and worsening the destabilization. Peak shifts and emergence (800–1200 cm^−1^) in infrared spectra confirmed that BCP and WP functional groups were altered after mixing and heating via electrostatic, hydrophobic, and hydrogen bonding interactions. This study demonstrated that appropriate processing conditions can positively impact anthocyanin-bound pectin–protein interactions.

## 1. Introduction

The interaction between polysaccharides, proteins, and polyphenols occurs in various food systems and is of great interest to the food industry as it affects the stability [1,2], appearance [3,4], texture [2,5], and taste of food [6,7]. The components (polysaccharides, proteins, and polyphenols) that interact may be from natural ingredients such as milk, egg, flour, and fruit puree; or isolated functional ingredients such as whey protein (WP), plant extracts, gelatine, and pectin. These ingredients may interact with each other through covalent and/or non-covalent bonds. For instance, polysaccharide–protein complexes that are prepared via electrostatic or covalent interaction can be used as a stable polyphenol delivery system to improve the incorporation of beneficial polyphenols in food [8,9]. Covalent interaction is highly specific to certain biopolymers and polyphenols, and it tends to be stable against pH and ionic changes. In contrast, non-covalent interactions can be affected by pH and ionic changes. The types of non-covalent interactions that can happen between the interactive components are (i) electrostatic interaction, (ii) hydrophobic interaction, and (iii) hydrogen bonding (H-bonding), although usually a multitude of interactive forces is responsible for the association of different functional groups of the components, and they have been discussed in various review papers [10,11,12,13,14,15,16].

In a binary polysaccharide–protein system, an anionic pectin is able to complex with WP via electrostatic interactions as a result of opposite charges between the carboxyl groups of pectin (negative) and the amino groups of protein (positive) [17,18,19,20,21,22]. However, the interaction depends heavily on the environmental pH as protein is amphoteric [23] while pectin can lose its negative charges at pH below its p*K*_a_ value [24]. Unlike the pectin-WP system, a polyphenol–protein system between anthocyanin (ACN) and WP is poorly documented, especially at the molecular level. The ACN may associate with WP via various modes of interaction depending on the structure of the ACN that changes according to its environmental pH. Generally, at very low pH (pH ≤ 1) the pyrylium ring of the ACN carries a positive charge and is known as the flavylium cation—a stable form of ACN that is red in color. Above pH 1, the equilibrium will shift to fewer flavylium cations due to the deprotonation of the pyrylium ring forming more quinoidal base ACN that is blue in color. In a slightly acidic medium, nucleophilic attack by water on the aglycone unit will occur forming a colorless ACN known as the carbinol pseudobase [25,26,27]. Due to the structure of ACN, it is believed that at different pH levels, ACN exhibits different biopolymer affinities where some reports have shown that flavylium cation binds more to biopolymers in comparison to carbinol pseudobase, especially when present at high concentration [16,28,29,30].

In addition to pH effects, heating also plays an important role in pectin–WP and ACN–WP interactions because the process of manufacturing food usually involves heat treatment. It is well known that heating at temperatures >60 °C causes the unfolding of the globular WP and exposure of its hydrophobic and charged amino acids (AA) [31,32,33], which increase the binding sites for pectin–WP and ACN–WP interactions. Furthermore, heating can alter the structure of ACNs to chalcones, which can further develop into the derivatives of aldehyde or benzoic acid [25].

Typically, a food system such as a fruit and milk-based beverage contains several key interactive components, like the polysaccharides and polyphenols from the fruit puree or juice, and the proteins from the milk. Hence, it is useful to study these components as a mixed system because the information gained can be used in managing the stability of these beverage systems. It has been reported that the capacity of polyphenols—grape seed extract, hibiscus extract, tannic acid, and catechin—to precipitate WP was reduced significantly when the WP was complexed to apple pectin, indicating that the associated pectin provided the protein with some stability against precipitation [1]. Many researchers have worked on similar interaction work following the study above, by exploring the interaction between other types of polysaccharides, proteins, and polyphenols for various research purposes [8,34,35,36,37,38]. Undeniably, these studies contributed useful insights to the current field of work, but the sole use of isolated ingredients may not be comparable to the use of a natural ingredient. Thus, a less refined composite material like the one used in the present work may offer a different approach. A recent study by Chevalier, Rioux, Angers and Turgeon [2] found that a blueberry pectic material (containing pectin, protein, and polyphenols) that was extracted from blueberry puree was able to complex with WP, and attributed the complexation to electrostatic interaction between the pectic and protein component. However, investigation into the influence of blueberry polyphenols on the blueberry pectin–protein complexes was not pursued as the focus was given to the study of rheological and textural properties of a blueberry–WP smoothie drink [2]. In another study, protein–polyphenol mesostructures were formed by complexing WP isolate with berry juice concentrates that were rich in polyphenols (mainly ACNs) and the authors attributed the complexation to protein–polyphenol interaction only, without addressing the possible interaction with the pectin in the juice concentrates [39].

The research gap from the above publications presented a bridging opportunity for a polysaccharide, protein, and polyphenol interaction study. Thus, a study between a composite pectic material known as the ACN-bound blackcurrant pectin (BCP) and WP was carried out as it allowed us to address the combined effect of pectin and ACNs when complexed with WP. The BCP was isolated from the juice of blackcurrant fruit via ethanol precipitation and dialysis at 4 °C and it is packed with bound ACNs (3.9% *w*/*w*) of which cyanidin 3-*O*-rutinoside (45%) and delphinidin 3-*O*-rutinoside (41%) are the two dominant ACNs [24]. Unlike the BCP, commercially available apple and citrus pectin have very low, or no polyphenols due to their extensive and harsher purification processes—acidic extraction between pH 1 and pH 3 at 50 °C to 90 °C—which may have removed and decreased their phenolic compounds [40,41]. In addition to bound ACNs, the BCP also contains some bound protein (4.8% *w*/*w*) and bound calcium (2.2% *w*/*w*) and has been recently characterized by our group [24].

We hypothesize that the impact of bound ACNs on the complexation between BCP and WP can be studied through pH influence (pH 3.5 and pH 4.5) and heat treatment (85 °C for 15 min). The two pH points were chosen based on: (i) the pH range of acidified milk drinks, which is between 3.6 to 4.6 [42], and (ii) the equilibrium of ACN structures at the two pH points—more flavylium cations at pH 3.5 and less flavylium cations at pH 4.5. The current work involved: (i) preparation of BCP-WP mixtures at various conditions—pH 3.5 and pH 4.5, without and with heating, and different heating sequences (mixed-heated vs. heated-mixed); (ii) stability, particle size and zeta-potential measurements of the mixtures; (iii) determination of BCP in the sedimented fraction of the mixtures; and (iv) investigation into the changes that occur in the functional groups of BCP and WP using Fourier Transform Infrared (FTIR) spectroscopy.

## 2. Materials and Methods

### 2.1. Materials

A dialyzed high methoxyl BCP (65.2 ± 10.2%) was isolated from blackcurrant juice and prepared according to the method described in our earlier work [24]. The blackcurrant juice was obtained by centrifuging seedless blackcurrant puree BLC5100 (Juice Products New Zealand Limited, Timaru, New Zealand) at 4618× *g* for 15 min at 20 °C. The composition of the BCP determined by proximate analysis can be obtained from our earlier publication [24].

The WP powder (Whey Protein Isolate 895) containing 94.2% *w*/*w* of protein was obtained from Fonterra, Auckland, New Zealand. It is noteworthy that protein aggregates can affect the size distribution of complexes that are formed via electrostatic interaction [43]. Therefore, aggregate-reduced WP was prepared based on Schmitt, Sanchez, Despond, Renard, Thomas and Hardy [43] method with some modifications. The protein powder was first hydrated with distilled water (20% *w*/*w*) and left stirring overnight at 4 °C. The pH of the solution was adjusted to pH 4.5 using 3 M and 6 M hydrochloric acid. Aggregate removal was carried out by high-speed ultra-centrifugation (78,702× *g* for 1 h at 20 °C) using a Sorvall WX Ultra 100, T-865 motor (Thermo Fisher Scientific Inc., Waltham, MA, USA). The supernatant was then carefully separated from the sediment, filtered using a 0.45 µm Durapore hydrophilic polyvinylidene fluoride vacuum filter (Merck Group, Darmstadt, Germany) and finally freeze-dried. The freeze-dried material contained 89.8% *w*/*w* of protein on dry weight basis.

Analytical grade D-(+)-galacturonic acid (GalUA) monohydrate (Sigma-Aldrich, St. Louis, MI, USA) and 3-phenylphenol (Sigma-Aldrich, St. Louis, MI, USA) were used as the standard and colorimetric reagent in GalUA analysis, respectively. Pure HPLC grade of cyanidin 3-*O*-rutinoside chloride and delphinidin 3-*O*-rutinoside chloride (Extrasynthese, Lyon, France) were used in zeta-potential measurements and FTIR analyses. All other chemicals were also of analytical grade and purchased from local companies. Chemical, stock, and buffer solutions were prepared using Milli-Q^®^ water (Merck Group, Darmstadt, Germany) and stored in dust-free bottles.

### 2.2. Complexation of Blackcurrant Pectin and Whey Protein

In the current study, the chosen concentrations of BCP and WP were similar to those previously used by Sperber, Schols, Cohen Stuart, Norde and Voragen [23]. Pectin and protein stocks (0.5% *w*/*v*) were prepared using distilled water and hydrated overnight at 4 °C under gentle stirring. Mixtures were kept in dust-free opaque bottles to avoid interference from dust particles during size measurements and to minimise light degradation of the ACNs. Appropriate amounts of BCP and WP stocks were measured to achieve a final concentration of 0.02% *w*/*v* BCP and a pectin:protein ratio of 1:1. Pectin concentration in BCP and protein concentration in the aggregate-reduced WP were calculated based on the amount of total carbohydrates (78% *w*/*w*) and protein (90% *w*/*w*) contained in the powders, respectively.

**Figure 1:**
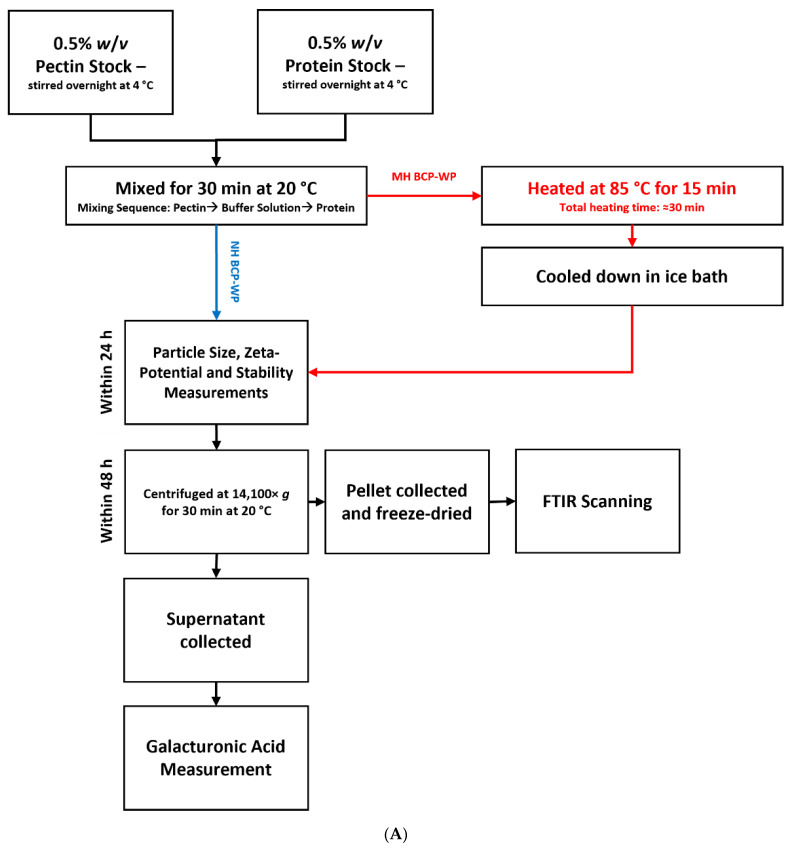

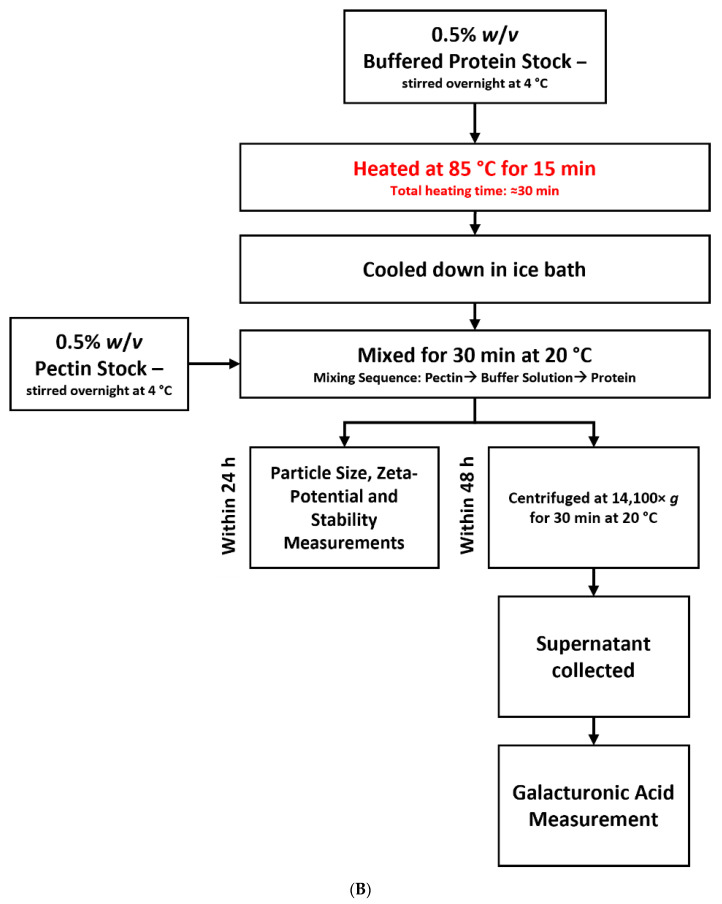
(**A**) Preparation steps for non-heated (NH) and mixed-heated (MH) blackcurrant pectin–whey protein (BCP-WP) mixtures and the measurement techniques used. (**B**) Preparation steps for heated-mixed blackcurrant pectin–whey protein mixture and the measurement techniques used.

Firstly, the BCP stock solution was added to the pH 3.5 sodium citrate buffer (1 mM) or pH 4.5 sodium acetate buffer (1 mM). Under gentle stirring, the protein stock was then added to the buffered pectin solution and then mixed at 20 °C for 30 min. This mixture is referred as the **“non-heated BCP-WP” (NH BCP-WP)** system (Figure 1A). For a **“mixed-heated BCP-WP” (MH BCP-WP) system** (Figure 1A), the mixture was heated at 85 °C for 15 min to achieve ≥90% protein denaturation [32] and then cooled down in an ice bath. Preparation of the **“heated-mixed BCP-WP” (HM BCP-WP)** system required the 0.5% *w*/*v* protein stock to be hydrated in buffered solution (Figure 1B) instead of distilled water. The buffered protein stock was then heated at 85 °C for 15 min and cooled down in an ice bath before adding it to the buffered pectin solution.

### 2.3. Stability Measurement

The stability of the mixtures was qualitatively determined by a Turbiscan Lab (Formulaction, Toulouse, France) where a near-infrared LED (λ_air_ = 880 nm) was used as the light source. The equipment measured two components: (i) the light transmitted through the mixture and (ii) the light backscattered by the mixture, at regular intervals over the height of the sample (30 mm). A clear cylindrical glass cell was used to contain the sample (4 mL) immediately after the sample was prepared, and data were collected every 30 s for 24 h at 20 °C. The Turbiscan Stability Index (TSI) is a relative number that evaluates the stability of the mixtures, calculated by the software TurbiSoft-Lab version 2.3 (Formulaction, Toulouse, France) by combining all variations from the backscattering and transmission data over the entire height of the sample measured. The TSI values are reported as the mean of two readings.

### 2.4. Particle Size and Zeta-Potential Measurements

Particle size (Z-average size) and zeta-potential of the controls (BCP and WP) and their mixtures were determined using a Zetasizer Pro (Malvern Panalytical Ltd., Malvern, UK). A low volume cuvette (ZEN0040) and folded capillary cell DTS1070 (Malvern Panalytical Ltd., Malvern, UK) were used for their measurements, respectively. Sample filtration was not performed prior to size measurement to avoid exclusion of the formed complexes. The zeta-potential values of ACN mix containing cyanidin 3-*O*-rutinoside (52%) and delphinidin 3-*O*-rutinosides (48%) were also determined by preparing a stock solution (0.173% *w*/*v*) and diluting it to 9 × 10^−4^% *w*/*v* using either a pre-chilled pH 3.5 sodium citrate (1 mM) or pH 4.5 sodium acetate (1 mM) buffer. The ACN solutions were prepared fresh in a dark room and kept in opaque bottles to minimise light degradation of the ACNs. Three measurement readings were taken for each sample at 20 °C. The Z-average and zeta-potential values are reported as the mean and standard deviation of at least two readings.

### 2.5. Quantification of Galacturonic Acid

Uronic acid was determined by Blumenkrantz and Asboe-Hansen [44] colorimetric method that was modified to fit a 96-well microplate [45]. Five GalUA solutions (40, 80, 120, 160, and 200 μg/mL) were prepared for constructing a standard curve and distilled water was used as a blank. The mixtures were centrifuged after 24 h (14,100× *g* for 30 min at 20 °C) and the supernatant was separated from the pellet. Centrifugation of the pectin control was also carried out, but no sedimentation was observed. The amount of GalUA in the supernatants was measured (regarded as the soluble complexes) and the percentage of GalUA in the pellet (regarded as insoluble complexes) was calculated by difference, based on the initial amount of GalUA in BCP. The assay was carried out twice and the samples were each measured in triplicate. The amount of uronic acid is reported as % (*w*/*w*) of GalUA equivalents and the mean and standard deviation of three readings.

### 2.6. Fourier Transform Infrared Spectroscopy

Pellets (insoluble complexes) obtained after centrifugation of the mixtures were freeze-dried and placed on an iD7 ATR (Thermo Fisher Scientific Inc., Waltham, MA, USA) diamond stage that was attached to a Nicolet™ iS™ 5 FTIR Spectrometer (Thermo Fisher Scientific Inc., Waltham, MA, USA). Since no sedimentation was observed in the BCP and WP controls at both pH values, dried powders of the controls were used for the scanning. In addition, the dried form of cyanidin 3-*O*-rutinoside chloride and delphinidin 3-*O*-rutinoside chloride were also scanned for comparison with BCP. The FTIR scanning was done at 20 °C and replicated twice for each sample (*n* = 2)—controls and freeze-dried insoluble fractions—because the recorded spectra between the duplicated scans and samples were highly reproducible. The infrared spectra were recorded within the range of 400–4000 cm^−1^ and band positions were identified by the Omnic™ Spectra software (Thermo Fisher Scientific Inc., Waltham, MA, USA). The results were averaged, normalised, and plotted using SigmaPlot 14.5 (Systat Software, Inc., Chicago, IL, USA).

### 2.7. Statistical Analysis

Statistical analyses were performed using SigmaPlot 14.5 (Systat Software, Inc., Chicago, IL, USA). A comparison between samples and controls was carried out using the One-Way Analysis of Variance. Values of *p* < 0.05 were considered statistically significant and Tukey’s multiple comparisons test was used to identify significant differences among the different samples.

## 3. Results and Discussion

### 3.1. Visual Appearance and Stability of Blackcurrant Pectin-Whey Protein Mixtures

**Figure 2:**
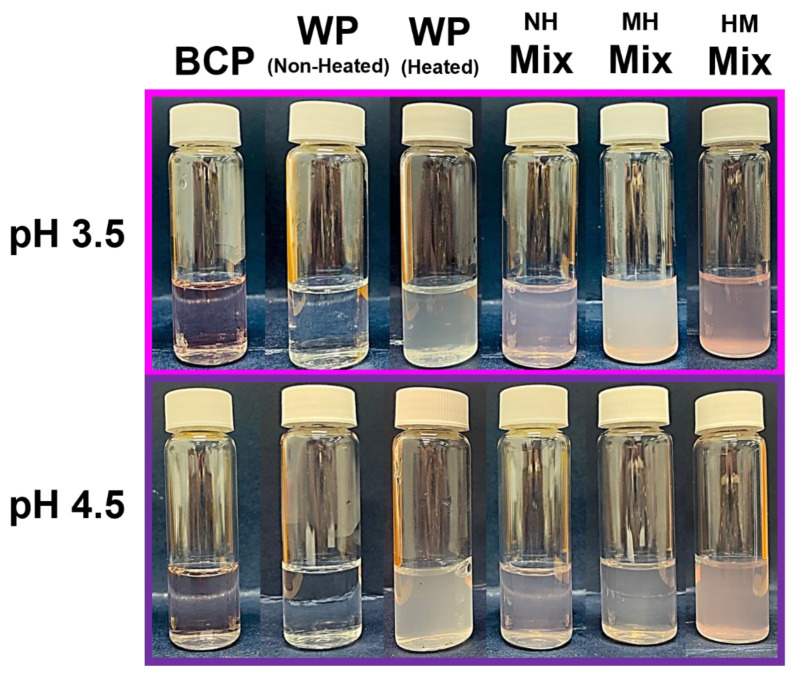
Blackcurrant pectin (BCP), whey protein (WP), and their mixtures (NH, non-heated; MH, mixed-heated; HM, heated-mixed) at pH 3.5 and pH 4.5.

Despite containing equal amounts (0.01 mg/mL) and types of ACNs (delphinidin 3-*O*-glucoside, delphinidin 3-*O*-rutinoside, cyanidin 3-*O*-glucoside and cyanidin 3-*O*-rutinoside) [24], color variation was observed between the two BCP controls at pH 3.5 (vivid pink) and pH 4.5 (pale purple) (Figure 2). This is likely due to the slight change in the molecular structure of the ACNs as their color depends on several factors: (i) the number of hydroxyl groups on the aglycone units, (ii) the presence or absence of methylation and glycosylation, and (iii) the amount of the positive charges found at the pyrylium ring [26,46]. The BCP control at pH 3.5 appeared pink because a proportion of the ACNs present were in the flavylium cationic form (red) and since water was present, some of the ACNs might also exist as the carbinol pseudobase (colorless) form. In contrast, the BCP control at pH 4.5 appeared to be pale purple because the ACNs were mainly in quinoidal base (blue) and carbinol pseudobase (colorless) form. A similar color variation was also observed in the NH and HM BCP-WP mixtures.

Regardless of the environmental pH, both the BCP and non-heated WP controls were transparent. When the two materials were mixed, they complexed with each other causing the mixtures to be turbid (NH mixtures), indicative of possible pectin–protein interactions. Applying heat caused the color of the MH mixtures to fade away. This is likely because the high heat (85 °C) can irreversibly change the structures of the ACNs by opening the pyrylium ring to form chalcones, which are colorless [25,47]. Further thermal degradation will then ensue, separating the ACNs from their sugar glycosyl molecules [25] and permanently disabling the ability of ACN to generate color. The turbid state of the heated WP control in Figure 2 clearly indicates that protein denaturation and protein–protein interactions had already occurred during the initial heating. Therefore, the turbidity observed in both HM mixtures might be contributed to by the turbidity of the heated WP control and the complexes from pectin–protein interactions. Since, heating was applied only to the protein component, the HM samples were able to retain the color of the ACNs.

**Figure 3:**
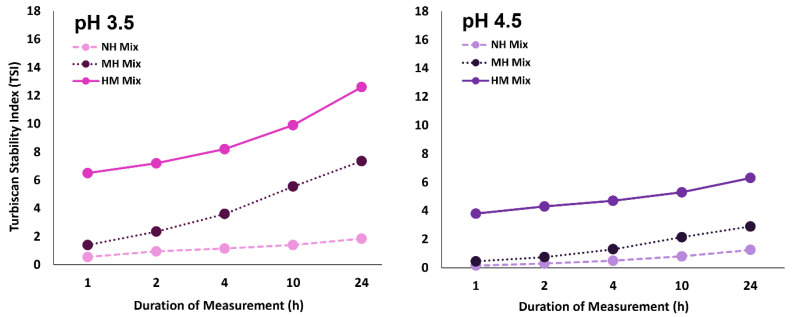
Turbiscan Stability Index (TSI) of blackcurrant pectin–whey protein mixtures (NH, non-heated; MH, mixed-heated; HM, heated-mixed) at pH 3.5 and pH 4.5 measured across 24-h timeframe.

Visually, it was difficult to differentiate the turbidity of pH 3.5 and pH 4.5 NH mixtures, particularly when they had comparable low TSI values (Figure 2 and Figure 3, respectively). However, when heated at pH 3.5, the destabilization of MH mixture increased drastically, matching its high turbidity shown in Figure 2. In contrast, the effect of heat on pH 4.5 MH mixture was less impactful. Heating increased the TSI value of pH 4.5 MH mixture slightly, but it did not affect its weak destabilized state nor its turbidity as it remained visually comparable to its NH counterpart. This could suggest that the heat treatment caused the increase in BCP-WP interactions at pH 3.5, but not so much at pH 4.5. On the other hand, the heating sequence (MH vs. HM mixtures) produced mixtures with very different stability. Heating WP separately before mixing with the BCP (HM mixture) led to a much greater instability at both pH levels, and at pH 3.5, the extent of turbidity was substantially worse. Heating the pectin–protein mixture (as opposed to heating the protein before mixing) seemed to improve the stability of the complexes substantially.

### 3.2. Particle Size and Zeta-Potential of Blackcurrant Pectin-Whey Protein Complexes

**Figure 4:**
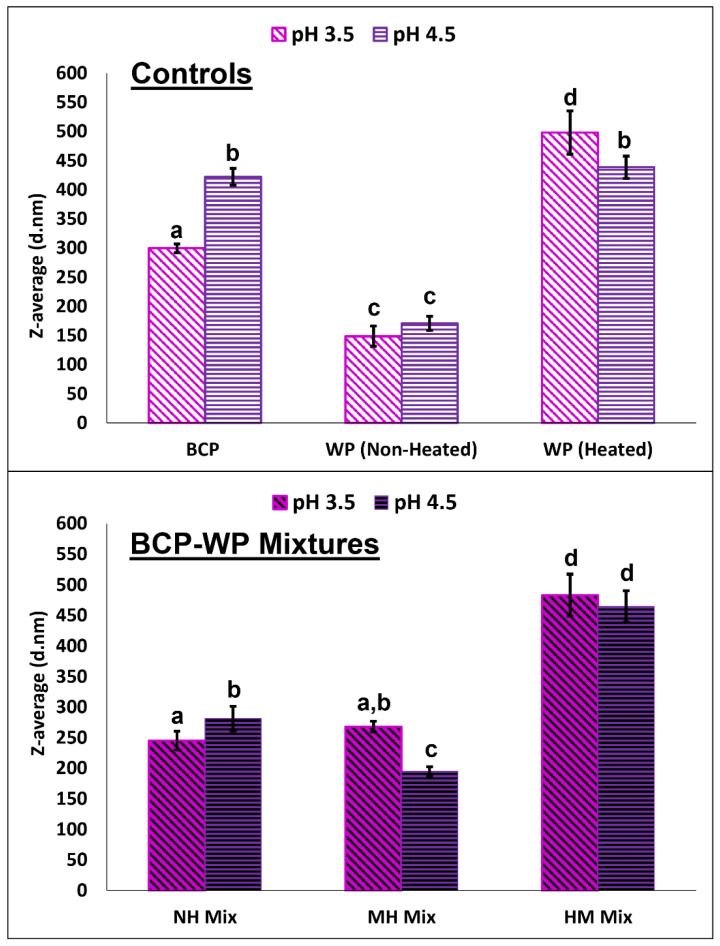
Particle size (nm, Z-averages mean ± standard deviation) of controls (BCP, blackcurrant pectin; WP, whey protein) and their mixtures (NH, non-heated; MH, mixed-heated; HM, heated-mixed) at pH 3.5 and pH 4.5. Means within a group are not significantly different if the letters (a–d) are the same.

**Figure 5:**
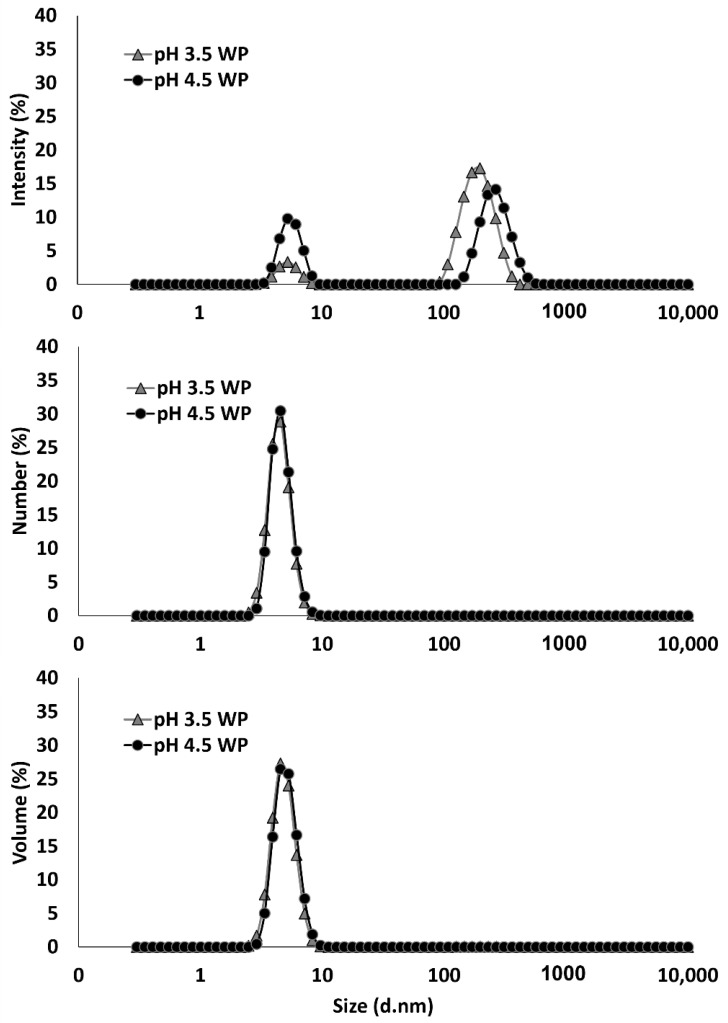
Average size distribution of aggregate-reduced whey protein (WP) at pH 3.5 and pH 4.5 based on intensity-, number-, and volume-weighting mechanisms.

In order to elucidate the trends seen on the turbidity and stability of the pH 3.5 and pH 4.5 BCP-WP mixtures, data on particle size and zeta-potential were recorded. Figure 4 shows the Z-average size of the controls (BCP and WP) and their mixtures (NH, MH, HM) at pH 3.5 and pH 4.5. The particle size of the BCP was significantly smaller at pH 3.5 (300 nm) than at pH 4.5 (422 nm). This is likely owing to weaker BCP intramolecular repulsion (less negative charge) at pH 3.5 as the pH is closer to the BCP p*K*_a_ of 1.7 [24,48]. The Z-average values for the non-heated WP controls at pH 3.5 and pH 4.5 were not significantly different (149 nm at pH 3.5 and 171 nm at pH 4.5), and although the Z-average size (based on scattering light intensity) were >100 nm, based on the number and volume distributions of both aggregate-reduced WPs (Figure 5), the majority of the protein particles that are scattering the light and interacting with pectin are very small, falling within the 4.5–5.1 nm range.

At both pH levels, the heat treatment increased the particle size of the WP considerably (Figure 4). It is well known that heat treatment of 85 °C unfolds globular proteins allowing aggregation via disulphide bonds and non-covalent bonds like hydrophobic and H-bonding interactions, significantly increasing their particle size [49,50]. However, the aggregate size was significantly bigger at pH 3.5 (498 nm) as compared to pH 4.5 (439 nm). Spiegel and Huss [50] also reported bigger aggregate size when WP was heated at a pH lower than pH 4.5. A likely explanation is that during heating at pH 3.5, the greater positive net charge of the WP provides the dominant β-lactoglobulin (β-lg) with more intramolecular repulsion that facilitates its unfolding [49] and subsequent aggregation with α-lactalbumin and bovine serum albumin (BSA). In contrast, the less positive net charge of the WP at pH 4.5 weakens the intramolecular repulsion of β-lg and tightens its protein conformation, subsequently reducing the degree of unfolding and the resultant Z-average size of heated WP.

Regarding the size of complexes (Figure 4), without heat treatment, the NH complexes at pH 3.5 (245 nm) and pH 4.5 (281 nm) had smaller particle sizes than their BCP controls (300 nm and 422 nm at pH 3.5 and pH 4.5, respectively). The size reduction can be attributed to the association of protein to the BCP, which reduced the intramolecular repulsion and compacted the conformation of BCP [23]. The complexes at pH 3.5 (245 nm) were significantly smaller than those at pH 4.5 (281 nm), but the Z-average size needs to be interpreted in conjunction with their particle size distributions as the presence of large particles can affect the reported Z-average size, and this will be discussed in the next section.

When heating was introduced to the complexed BCP and WP, the MH mixture at pH 4.5 experienced a significant size decrease from 281 nm to 195 nm without and with heat, respectively, yet no significant change in size was observed at pH 3.5—245 nm without heat vs. 268 nm with heat (Figure 4). Between the MH mixtures, pH 3.5 (268 nm) seemed to form significantly larger complexes than pH 4.5 (195 nm) and this comparison will be confirmed via their particle size distributions in the following section. On the contrary, if the heating sequence was changed to HM, it produced complexes—483 nm at pH 3.5 and 465 nm at pH 4.5—that were significantly larger than the NH and MH complexes. The HM complexes at both pH levels (483 nm at pH 3.5 and 465 nm at pH 4.5) were not significantly different, and they seemed to follow the size of their respective aggregated WP controls (pH 3.5: 498 nm, pH 4.5: 439 nm).

**Figure 6:**
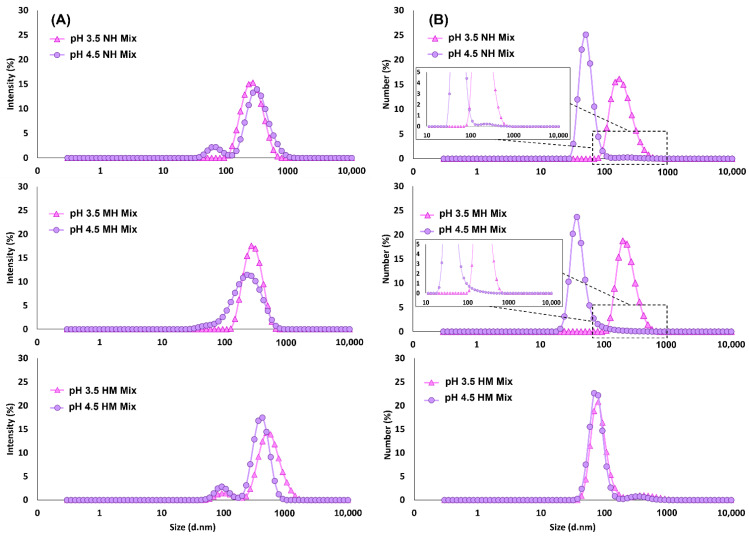
Average size distribution of blackcurrant pectin–whey protein mixtures (NH, non-heated; MH, mixed-heated; HM, heated-mixed) at pH 3.5 and pH 4.5 based on (**A**) intensity and (**B**) number weighting mechanisms.

Figure 6 shows the particle size distribution of NH, MH, and HM mixtures at pH 3.5 and pH 4.5. Based on light-scattering intensity (Figure 6A), the NH system at pH 4.5 has two populations of complexes (small peak: ≈68 nm and large peak: ≈346 nm), whereas at pH 3.5 only one population is seen (≈276 nm). According to the number distribution of NH mixtures (Figure 6B), the majority (99%) of the complexes at pH 4.5 (≈53 nm) were much smaller than the complexes at pH 3.5 (≈199 nm), however, a small number of large complexes (≈261 nm) was detected at pH 4.5, as shown in the inset figure, represented by the minor peak. Large particles can affect the Z-average size of a system because they scatter light with greater intensity that overshadows the intensity from the smaller particles. This renders the calculated Z-average size to be less precise as it is not depicting the actual population of the complexes. This was the case for the pH 4.5 NH system where its Z-average size (281 nm) was larger than that of pH 3.5 (245 nm), yet, based on number distribution, the majority of its complexes were smaller (<100 nm) than the pH 3.5 complexes (>100 nm). A likely reason behind the larger NH complexes at pH 3.5 is that they may be associated with more WP molecules than those at pH 4.5.

As mentioned previously, heating the NH mixtures changed the Z-average size of MH complexes at pH 4.5 but not at pH 3.5, and this size trend is also reflected in their intensity and number distribution shown in Figure 6A,B. The small peak, which symbolizes the large complexes, seen in the inset figure of NH mixture at pH 4.5 had disappeared after heat was applied (inset figure of MH mixture at pH 4.5), indicating that the heat had altered or broken up the large complexes at pH 4.5 (Figure 6B). The number distribution plot also confirmed that the MH complexes at pH 3.5 were larger (≈237 nm) than those at pH 4.5 (≈44 nm), matching the Z-average size trend. As mentioned previously, a possible reason behind the larger MH complexes at pH 3.5 is that they may be associated with more WP molecules than those at pH 4.5, which corresponded with the more turbid pH 3.5 MH mixture shown in Figure 2.

Based on Figure 6A of HM mixtures, (i) the presence of an additional peak in pH 3.5 mixture, (ii) the increase of peak intensity in pH 4.5 mixture, and (iii) the shifting of overall peaks to the right side of the distribution plot indicates that there was a major change in the particle size of the HM mixtures. The number distribution plot revealed that there was an increase in particle size and the population of large aggregates—≈79 nm (96%) and ≈359 nm (4%) at pH 3.5, and ≈86 nm (93%) and ≈483 nm (7%) at pH 4.5, in comparison to the NH systems. The increase in size and population of large aggregates is likely to be the reason for the instability of the HM mixtures shown in Figure 3.

The polydispersity index (PDI)—calculated based on intensity distribution—of the NH, MH, and HM mixtures at pH 3.5 and pH 4.5, is shown in Table 1. Mixtures that were not heated seemed to be medium polydisperse (0.1 to 0.4) with pH 4.5 having significantly higher PDI (0.39 ± 0.05), possibly due to the presence of two population peaks in the pH 4.5 NH system, as shown in Figure 6A. When heat was applied, it caused the size distribution of the MH mixtures to significantly narrow down because the heat could have compacted the complexes of pectin and its associated proteins [51], reducing the PDI to 0.12 ± 0.03 and 0.26 ± 0.01 at pH 3.5 and pH 4.5, respectively while maintaining their medium polydispersity. The described changes can be seen in Figure 6A because the peak in pH 3.5 MH mixture was narrower than its NH counterpart and that the two population peaks seen in pH 4.5 NH mixture were reduced to just a single peak after heating, thus the reduced PDI. In contrast to the MH mixtures, the PDI of both HM mixtures were significantly higher (0.52 ± 0.03 and 0.37 ± 0.03 at pH 3.5 and pH 4.5, respectively), probably due to a wider population of complexes that consisted of larger aggregates.

**Figure 7:**
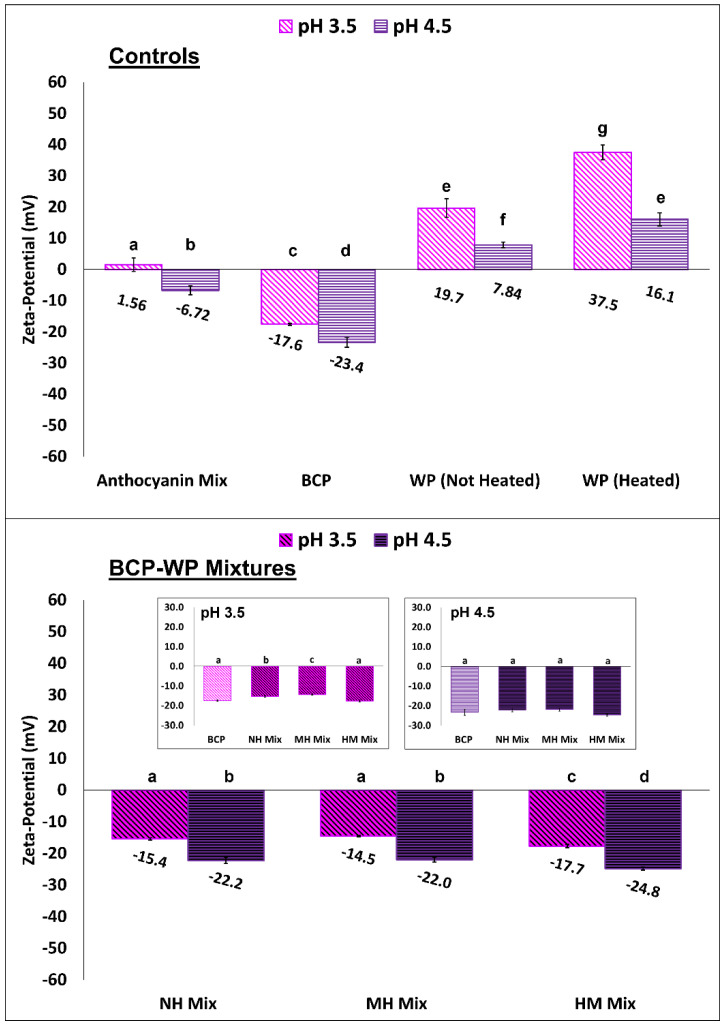
Zeta-potential (mV, mean ± standard deviation) of controls—anthocyanin mix (cyanidin 3-*O*-rutinoside and delphinidin 3-*O*-rutinoside), blackcurrant pectin (BCP), and whey protein (WP)—and their mixtures (NH, non-heated; MH, mixed-heated; HM, heated-mixed) at pH 3.5 and pH 4.5. Means within a group are not significantly different if the letters (a–g) are the same.

Figure 7 shows the zeta-potential values of ACN mix (cyanidin 3-*O*-rutinoside and delphinidin 3-*O*-rutinoside), BCP, WP, and their mixtures (NH, MH, HM) at both pH levels. The concentration of the ACN mix (9 × 10^−4^% *w*/*v*) represented the amount of ACNs present in the BCP (0.02% *w*/*v*) of the pectin–protein mixtures (Figure 7). The counter ions contributed to by the ACNs at pH 3.5 (+1.56 mV) and pH 4.5 (−6.72 mV) were not significant for the BCP-WP mixtures, and the slight negative charges observed at pH 4.5 were probably contributed by the chloride ions. At pH 3.5, the BCP control had a moderate amount of negative charge that significantly increased as the pH became less acidic—from −17.6 mV at pH 3.5 to −23.4 mV at pH 4.5—because as the pH is further away from its p*K*_a_ of 1.7, more carboxyl groups would be dissociated [24]. The WP controls had significantly different zeta-potential values at pH 3.5 (+19.7 mV) and pH 4.5 (+7.84 mV). At the p*I* of the WP (pH 5.3) [52], the protein carries charged basic amino groups (NH_3_^+^) and acidic carboxylate groups (COO^−^) at its backbone, and these functional groups can be deprotonated to NH_2_ or be protonated to COOH, respectively, depending on the pH of the system [53]. As the pH decreases further to pH 3.5, more COO^−^ groups are protonated to COOH, increasing the overall positive charge of the WP, thus, the higher zeta-potential at pH 3.5 as compared to pH 4.5. Heating the WP controls revealed hidden AAs that carry side chains with ionizable groups and thereby, increase the overall surface charge of the heated WPs (+37.5 mV and +16.1 mV at pH 3.5 and pH 4.5, respectively) as previously observed by Jiang, et al. [54] and Zhao and Xiao [55].

The zeta-potential plot of the NH, MH, and HM mixtures at pH 3.5 and pH 4.5 (Figure 7) shows that there was a minor decrease of surface charge in two of the mixtures (NH and MH), but not in the HM mixtures. At pH 3.5, the zeta-potential values of NH (−15.4 mV) and MH (−14.5 mV) mixtures were significantly different from the zeta-potential value of BCP (−17.6 mV, inset figure of Figure 7), indicating that the reduction of surface charge was likely to be caused by pectin–protein interactions. Yet at pH 4.5, the surface charges of NH, MH, and HM mixtures (−22.2 mV, −22.0 mV and −24.8 mV, respectively) were comparable to the pH 4.5 BCP (−23.4 mV, inset figure of Figure 7), suggesting that complexation at higher pH had a minor impact on the surface charge of BCP. Nevertheless, the zeta potential values of all the pectin–protein mixtures (NH, MH, HM) seemed to be governed by the BCP, irrespective of their pH levels and heat treatment. This might suggest that the excess BCP in the mixtures or unoccupied binding sites in the BCP rendered the overall mixed particles as negatively charged particles. Based on the surface charge of the complexes, systems at pH 3.5 and pH 4.5 can be considered as having relatively (±10–20 mV) and moderately (±20–30 mV) electrostatic stability, respectively [56].

Overall, NH and MH BCP-WP mixtures at pH 3.5 seemed to form larger complexes (>100 nm) than those at pH 4.5 (<100 nm), a probable indication that the interactive forces between BCP and WP at pH 3.5 are stronger, leading to more polymer association. Even though, the NH and MH complexes at both pH points were negatively charged, these aggregates at pH 4.5 had stronger electrostatic stability (>20 mV) than those at pH 3.5 (<20 mV). The weaker electrostatic stability at pH 3.5 was probably insufficient to keep the denser MH complexes suspended and stable as more WP was associated with it. Furthermore, the effect of heat on the turbidity of the MH mixture was also more pronounced in pH 3.5, coupling this observation with the large complexes size (>100 nm) and the weaker electrostatic stability (<20 mV) led to the overall instability of the pH 3.5 MH mixture.

### 3.3. Insoluble Complexes in Blackcurrant Pectin-Whey Protein Mixtures

**Figure 8:**
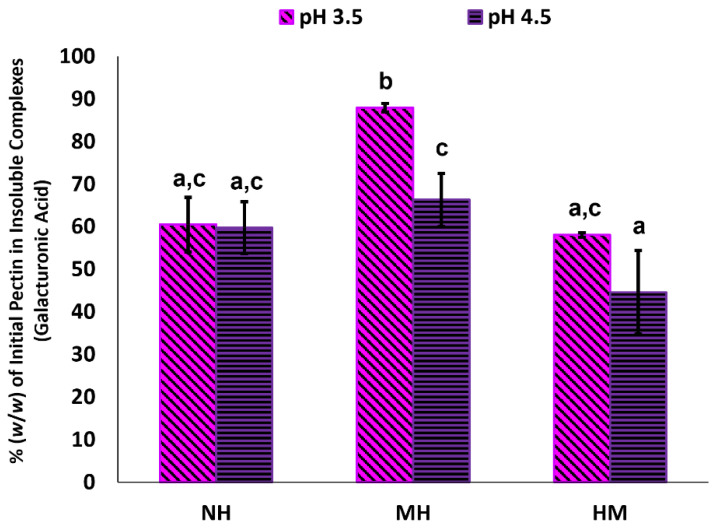
Percentage of the initial pectin (*w*/*w*, expressed as mean galacturonic acid ± standard deviation) found as part of the insoluble complexes of blackcurrant pectin–whey protein mixtures (NH, non-heated; MH, mixed-heated; HM, heated-mixed) at pH 3.5 and pH 4.5. Means with the same letter (a–c) are not significantly different.

Besides characterizing the complexes in the BCP-WP mixtures, it is also necessary to evaluate the sedimented insoluble fractions, thus this section reports on this. Figure 8 shows the amount of pectin detected in the insoluble complexes of NH, MH, and HM mixtures at pH 3.5 and pH 4.5. Insoluble complexes were captured as the fraction that sedimented during centrifugation; neither BCP nor WP at both pH levels showed any sign of sedimentation. Both insoluble fractions of NH mixtures had a comparable amount of pectin (61% and 60% *w*/*w* at pH 3.5 and pH 4.5, respectively). Despite that, the larger complexes of pH 3.5 NH mixture (≈199 nm)—seen in the number distribution of Figure 6B—may suggest that there is greater amount of WP associated with the pH 3.5 BCP, probably owing to the combined effect of (i) greater positive charge of WP at pH 3.5 (+19.7 mV) and (ii) the presence of more flavylium cations in the BCP. In contrast, the lower positive charge of WP (+7.84 mV) and the less flavylium cations at pH 4.5 could have led to lesser WP association and, therefore, the smaller NH complexes at pH 4.5 (≈53 nm based on Figure 6B).

When heat was applied to the pectin–protein mixtures, a significant increase of pectin in MH insoluble fraction was detected at pH 3.5 (88% *w*/*w* of the initial pectin), revealing the enhancement of complexation at this pH upon heating. The amplified complexation could be attributed to the interaction of pectic carboxyl groups and bound ACNs with the newly exposed AAs of unfolded WP. In addition to hydrophobic and H-bonding interactions, flavylium cations of pH 3.5 BCP might also interact with the WP electrostatically via AAs with acidic side chains (aspartic acid and glutamic acid), and this was likely because aspartic and glutamic acids are the two most abundant AAs of WP [57]. Furthermore, the comparable size of NH and MH complexes at pH 3.5 (245 nm and 268 nm, respectively in Figure 4) probably suggests that more WPs were associated with the pH 3.5 BCP, yet size difference was not observed because the heat had compacted the complexes, causing the particle size to be similar. On the contrary, heating at pH 4.5 was not enough to show the enhancement effect of bound ACNs as the increase of pectin in MH insoluble complexes (66% *w*/*w*) was not statistically different than its NH counterpart (60% *w*/*w*). This coincides with the significant size decrease observed between the NH and MH complexes at pH 4.5 (281 nm and 195 nm, respectively in Figure 4), suggesting that the lower positive charge of WP (+16.1 mV) and the lesser flavylium cations made the complexation between the pectin and protein at this pH to be less intense, while the heat compacted the complexes into smaller particles.

In comparison to the NH mixtures, mixing BCP with the heated WP—presumably, unfolded protein with interactive sides exposed—did not increase the amount of pectin in the insoluble complexes of HM mixtures at pH 3.5 (58% *w*/*w*) and pH 4.5 (45% *w*/*w*), and were regarded as not significantly different. The protein–protein interactions may have dominated when WP was heated before mixing, leading to a decrease of available binding sites for interaction with BCP.

**Figure 9:**
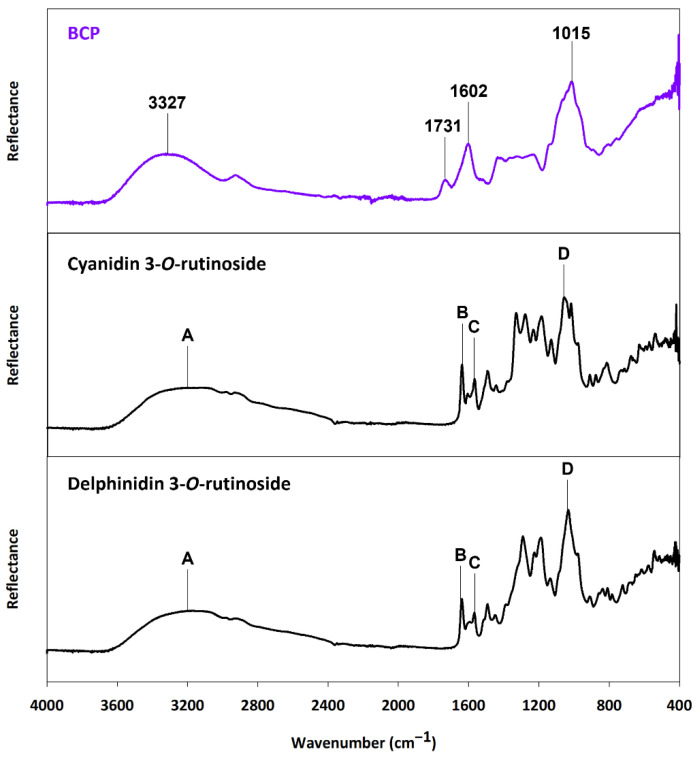
FTIR spectra of blackcurrant pectin (BCP) and the two major anthocyanins (cyanidin 3-*O*-rutinoside and delphinidin 3-*O*-rutinoside) found in blackcurrant. Peaks A to D represent the functional groups of anthocyanin that coincided with BCP peaks.

FTIR spectroscopy was used to identify the functional groups of blackcurrant ACNs, BCP, and WP, before comparing them against the insoluble fraction of the BCP-WP mixtures. The FTIR spectrum of BCP was first compared against the spectrum of the two major ACNs of blackcurrant (Figure 9). The overall broadness of BCP peaks is an indication that there are various intermolecular interactions happening within the BCP, and this is because the pectic material contained bound components like ACNs, proteins, and minerals [24]. Choong, et al. [58] reported a similar spectrum with a broad region around 2500–3800 cm^−1^ for *Hibiscus sabdariffa* (Roselle) plant extract that contained pectin and ACNs. The first broad peak of BCP (3327 cm^−1^) is comparable to the broad peak (Peak A) seen in both ACN spectra. The smoothness and broadness of the region between 2400 and 3600 cm^−1^ of the BCP are probably characteristic of an ACN-rich material. This region was assigned to: (1) the O-H stretch of carboxylic and phenolic groups of the pectin and ACNs, respectively, and (2) the C-H stretch of the alkyl and aromatic groups of the pectin and ACNs, respectively [59,60].

The second (1731 cm^−1^) and third (1602 cm^−1^) peaks of the BCP corresponded to the C=O stretching of the esterified and non-esterified carboxyl groups of the pectin chain, respectively [60,61]. This region coincided with the fingerprint region of the two ACNs, that represented the C=C stretch of alkenes and aromatic C-C groups (Peak B and Peak C, respectively) [59,62]. The final distinct peak (1015 cm^−1^) of the BCP between 800 and 1200 cm^−1^ might represent the various functional groups of the BCP components such as the C-O stretch of the carboxylic acids, ethers, esters, and alcohol groups of the pectin or its bound protein. Within that same region, both ACNs also showed a strong peak (Peak D) that could correspond to the C-C (stretches, bends, and torsions) backbone and the many C-O stretches and C-H bends of ACNs. Due to the overlapping region of interest, it was difficult to distinguish the peaks and make definitive assignments of the ACN functional groups. This was expected, as the BCP is a complex material, and therefore only approximate location of the ACN functional groups was used for the complexation study.

**Figure 10:**
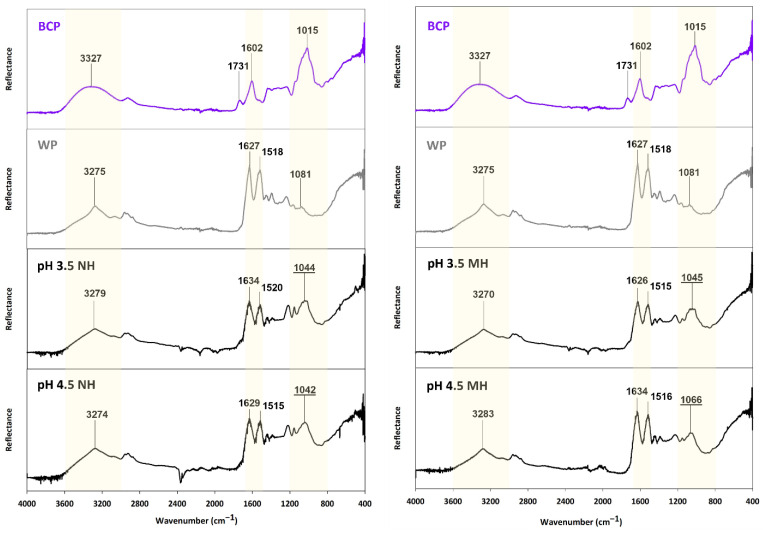
FTIR spectra of blackcurrant pectin (BCP), whey protein (WP), and the insoluble complexes of their mixtures (NH, non-heated; MH, mixed-heated; HM, heated-mixed) at pH 3.5 and pH 4.5.

Further study was carried out on NH and MH mixtures as they showed better dispersion stability. FTIR spectra were recorded for the insoluble complexes of the two systems to investigate the changes that occurred in the functional groups of the BCP and WP following their complexation. The WP spectrum was used as a reference because the spectrum of the sedimented fractions resembled the WP spectrum more than the BCP (Figure 10). Note that some of the spectra in Figure 10 show a carbon dioxide band (≈2340 cm^−1^) and artefacts from the ATR diamond between 2000 and 2400 cm^−1^.

Irrespective of the pH, peak shifts are seen throughout the spectrum of NH and MH insoluble complexes, indicating that the complexation between BCP and WP had affected their functional groups (Figure 10). The first broad peak of the sedimented fractions between 3000 and 3600 cm^−1^ shifted slightly away from the first reference peak of WP (3275 cm^−1^). Peak shift within that region is attributed to pectin and protein interacting electrostatically as the region signifies the polymeric interaction between the O-H groups of carboxylic acids—originating from BCP and WP—and the N-H groups of amides and amines that originate from the WP [60]. As mentioned earlier, the O-H groups of the bound ACNs were also assigned to this region [58,59,62]; however, based on these results, the extent of their contribution to the complexation is not clear due to the undistinguishable region.

Shifting of peaks is also detected on the two prong-like peaks of the sedimented fractions—between 1460 and 1680 cm^−1^. The two peaks (1627 and 1518 cm^−1^) were originally identified in the WP spectrum corresponding to the C=O stretching of amides and the N-H bend of amines of WP, respectively [59,60]. As mentioned previously, this region also coincided with the non-esterified C=O groups of BCP [61] and the C=C stretch of alkenes and aromatic C-C groups of bound ACNs [59,62]. Similar spectral shifts were observed in the complexation between ACN-free high methoxyl apple pectin and WP, signifying the impact of electrostatic interaction on the two prong-like peaks [60]. However, in this study, the presence of ACN aromatic groups in the same region means that other interactive forces such as H-bonding and hydrophobic interaction can also contribute to the peak shifts.

In addition to the spectral shifts, a new peak between 800 to 1200 cm^−1^ was also identified in the spectra of the sedimented fractions. The new peak coincided with the location of aliphatic amines of WP and various functional groups of the BCP (pectic and ACN components). Based on the nature of these functional groups, the new peak might be related to H-bonding and hydrophobic interaction, confirming that an additional type of interaction other than electrostatic had occurred. At pH 4.5, the spectrum of MH insoluble complexes shows that the new peak shifted to 1066 cm^−1^ as opposed to the rest of the insoluble complexes that peaked at 1042–1045 cm^−1^. This is likely due to the interaction of WP with thermally altered bound ACNs of BCP. The molecular structure of ACNs at pH 4.5 is more susceptible to thermal changes, therefore, thermal degradation accelerates at less acidic pH [63], producing derivatives of aldehyde or benzoic acid [25]. These compounds are likely to interact with the WP as they have an aromatic ring on their molecular structure, and their interaction with WP may have impacted the location of that new peak.

### 3.4. Overall Discussion: Possible Mechanism of Complexation between Blackcurrant Pectin and Whey Protein

**Figure 11:**
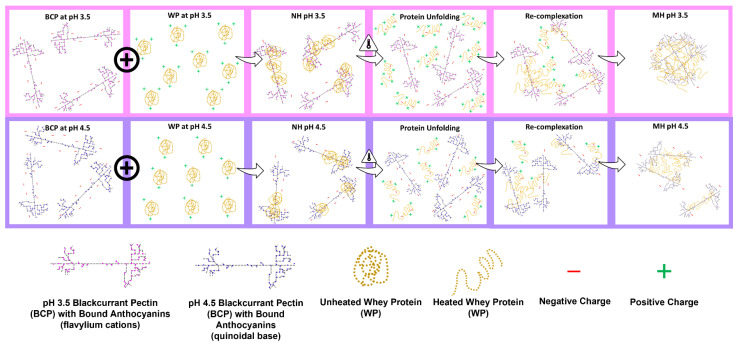
Schematic diagram of non-heated (NH) and mixed-heated (MH) blackcurrant pectin–whey protein mixtures at pH 3.5 and pH 4.5.

Based on the results described in previous sections, a schematic diagram in Figure 11 illustrates the possible NH and MH complexation of BCP and WP at pH 3.5 and pH 4.5. The first component of the diagram shows the negatively charged BCP and its bound ACNs at both pH levels. The BCP is negatively charged at pH 3.5 and its bound ACNs appear pink because the majority of the ACNs are protonated as the flavylium cation. When the pH increases to pH 4.5, the BCP becomes more negative and turns purple as the majority of its bound ACNs are deprotonated to the blue quinoidal base. The net charge of the WP increases as the pH moves away from the p*I* of the WP (pH 5.3) [30], therefore, the WP at pH 3.5 is more positively charged than at pH 4.5 as shown in Figure 11.

The complexation starts when the oppositely charged BCP and globular WP are mixed, forming complexes that carry net negative charges at both pH levels, probably due to excess or unoccupied binding sites of BCP. In the absence of heat, findings from FTIR indicate that the complexation occurs as a result of combinative interactions: (i) electrostatic interactions between the negatively charged BCP and positively charge WP, (ii) H-bonding of ACN hydroxyl groups with amino and hydroxyl groups of WP, as well as (iii) hydrophobic interactions between the ACNs and hydrophobic AAs such as tryptophan19 and tryptophan134 that can be found on the surface of β-lg and BSA, respectively. More WP may also associate with the BCP at pH 3.5 as the WP is more positively charged and there are more flavylium cations on the BCP resulting in larger complexes. At pH 4.5, the WP is less positively charged and less flavylium cations are available on the BCP; therefore, the complexes formed are generally smaller.

When the mixtures are heated at 85 °C, the complexes are likely to undergo a simultaneous restructuring of components, involving the partial unfolding of the WP and its re-complexation with the BCP via the mechanism described earlier before assuming the ‘core shell arrangement’ [64,65]. At pH 3.5, the intramolecular charges of the globular WP aid in the unfolding of its protein structures, exposing the hydrophobic AAs and other charged AAs that are once hidden. The newly exposed AAs can now act as available sites for the pectin, ACNs, and possibly the nearby soluble complexes to cross-link with, which eventually permit more pectin–protein and can–protein complexation to occur. The ACNs at pH 3.5 are most likely to interact with the exposed AAs via H-bonding, and hydrophobic and ionic interaction, depending on the side chains of the AAs [28], and H-bonding will eventually ensue to reinforce their associations [14]. The acidity of the system may have provided the BCP with flexibility [48], which makes it advantageous during the re-complexation process as it allows more contact between the binding sites. These occurrences proved that heating intensified the interaction between the BCP and WP at pH 3.5, causing the increase of turbidity (Figure 2) in the MH mixture and the increase of pectin percentage (Figure 8) in MH-insoluble complexes that adversely affected its stability (Figure 3). The lower percentage of pectin in MH-insoluble complexes at pH 4.5 may be owing to a combination of factors: (i) the lesser positive charges of WP, (ii) the unfolding resistance of the WP that may have reduced the exposure of concealed AAs, (iii) fewer binding sites of ACNs, and (iv) the reduced flexibility of the BCP. These reasonings correspond well with the turbidity and stability of the pH 4.5 MH system observed in Figure 2 and Figure 3.

## 4. Conclusions

The present study demonstrated that the bound ACNs of BCP can affect the complexation between BCP and WP. The complexation was increased at lower pH while in the presence of heat, probably because of the electrostatic interaction between cationic form of ACNs and the two dominant AAs of WP that carried acidic side chains. The overall stability of the NH and MH pH 4.5 mixtures was probably conferred by steric stabilization and their relatively higher net charges, which provided the complexes with some electrostatic stability. Remarkably, the stability of the pH 4.5 MH system that was comparable to its NH version could be attributed to the following reasons: (i) the lower amount of BCP-WP interactions that led to smaller particle sizes, (ii) the relatively low polydispersity, and (iii) the moderate electrostatic stability. The sequence of heating demonstrated a huge impact on the stability of the mixtures. Heating of WP at pH ≤ 4.5 produced aggregates that are not ideal for high protein beverages, especially if the mixture contains fruit or berry materials that are packed with polyphenols such as ACNs. In the absence of food stabilizers, the stability of the system will be adversely affected, particularly in a moderately acidic environment. Although definitive bands were not assigned due to overlapping regions of interest, findings from FTIR spectroscopy clearly showed that the complexation between BCP and WP had altered their functional groups and that electrostatic interaction is not the only mode of interaction between the ACN-bound BCP and WP. The emergence of a new peak in the spectrum of BCP-WP insoluble fractions strongly suggests that the additional mode of interaction could be related to H-bonding and hydrophobic interactions. Further work using FTIR spectroscopy will confirm the presence of BCP-WP soluble complexes, which is the subject of our next study, together with the quantification of protein in the soluble fractions. This will allow a better understanding of the distribution pattern of the components in both soluble and insoluble fractions.

## Figures and Tables

**Table 1:** Polydispersity index (mean ± standard deviation) of blackcurrant pectin–whey protein mixtures (NH, non-heated; MH, mixed-heated; HM, heated-mixed) at pH 3.5 and pH 4.5. Means with the same letter (a–d) are not significantly different.

	Polydispersity Index	
	pH 3.5	pH 4.5
**NH Mix**	0.21 ± 0.02 ^a^	0.39 ± 0.05 ^b^
**MH Mix**	0.12 ± 0.03 ^c^	0.26 ± 0.01 ^a^
**HM Mix**	0.52 ± 0.03 ^d^	0.37 ± 0.03 ^b^

## Data Availability

Not applicable.

## Sample Availability

Samples of the compounds are available from the authors.
